# Workplace violence against emergency health care workers: What Strategies do Workers use?

**DOI:** 10.1186/s12873-022-00621-9

**Published:** 2022-05-06

**Authors:** Evelien Spelten, Julia van Vuuren, Peter O’Meara, Brodie Thomas, Mathieu Grenier, Richard Ferron, Jennie Helmer, Gina Agarwal

**Affiliations:** 1 Violet Vines Marshman Research Centre, Rural Health School, La Trobe University, Melbourne, Australia; 2 Department of Paramedicine, Monash University, Melbourne, Australia; 3 County of Renfrew Paramedic Service, Pembroke, Canada; 4 Niagara Emergency Medical Services, Niagara Region, Niagara, Canada; 5 School of Population and Public Health, University of British Columbia, Vancouver, Canada; 6 British Columbia Emergency Health Services, Vancouver, Canada; 7 Department of Family Medicine, McMaster University, Hamilton, Canada; 8 Department of Health Research Methods, Evidence, and Impact, McMaster University, Hamilton, Canada

**Keywords:** Paramedicine, Emergency Medical Services, Occupational health, Violence, Workplace violence

## Abstract

**Background:**

Workplace violence by patients and bystanders against health care workers, is a major problem, for workers, organizations, patients, and society. It is estimated to affect up to 95% of health care workers. Emergency health care workers experience very high levels of workplace violence, with one study finding that paramedics had nearly triple the odds of experiencing physical and verbal violence.

Many interventions have been developed, ranging from zero-tolerance approaches to engaging with the violent perpetrator. Unfortunately, as a recent Cochrane review showed, there is no evidence that any of these interventions work in reducing or minimizing violence.

To design better interventions to prevent and minimize workplace violence, more information is needed on those strategies emergency health care workers currently use to prevent or minimize violence.

The objective of the study was to identify and discuss strategies used by prehospital emergency health care workers, in response to violence and aggression from patients and bystanders. Mapping the strategies used and their perceived usefulness will inform the development of tailored interventions to reduce the risk of serious harm to health care workers.

In this study the following research questions were addressed: (1) What strategies do prehospital emergency health care workers utilize against workplace violence from patients or bystanders? (2) What is their experience with these strategies?

**Methods:**

Five focus groups with paramedics and dispatchers were held at different urban and rural locations in Canada**.** The focus group responses were transcribed verbatim and analyzed using thematic analysis.

**Results:**

It became apparent that emergency healthcare workers use a variety of strategies when dealing with violent patients or bystanders. Most strategies, other than generic de-escalation techniques, reflect a reliance on the systems the workers work with and within.

**Conclusion:**

The study results support the move away from focusing on the individual worker, who is the victim, to a systems-based approach to help reduce and minimize violence against health care workers. For this to be effective, system-based strategies need to be implemented and supported in healthcare organizations and legitimized through professional bodies, unions, public policies, and regulations.

## Introduction

Workplace violence against health care workers, by patients and bystanders, is a major problem, for health care workers, organizations, patients, and society. It is estimated to affect up to 95% of health care workers [1–3]. Emergency health care workers experience very high levels of workplace violence, with one study finding that paramedics had nearly triple the odds of experiencing physical and verbal violence [4].

The World Health Organization (WHO) categorizes workplace violence into physical and psychological violence, which includes verbal violence [5]. In practice a distinction can be made between major incidents resulting in injury or death of the worker, and everyday violence. While there is general outrage when there is a major accident [6], the everyday violence from patients and bystanders (including name calling, spitting) does not get as much attention. Workplace violence against health care workers is unlikely to be eliminated, however, an achievable aim is to design and implement interventions that will reduce and minimize this violence and contribute to a safer work environment.

Many interventions have been developed, ranging from zero-tolerance approaches to engaging with the violent perpetrator. Unfortunately, as a recent Cochrane review showed, there is no evidence that any of these interventions work in reducing or minimizing workplace violence [3]. The focus of many interventions is on managing violent incidents, rather than preventing or minimizing them. This is evident in the almost universal training of health care workers in de-escalation techniques [7], indicating a one-size-fits-all approach to violence [8].

A one-size-fits-all approach has its limitations. Violence from patients and bystanders varies, depending on the type of perpetrator [9, 10] or the environment in which it occurs. For example, a standard hospital ward provides a much more controlled environment than the work environment of a (community) paramedic or an emergency department [3]. Prehospital emergency care workers stand out because of the nature of their work environment which is uncontrolled and often involves acute situations. They have a patient population that is more heterogeneous than a mental health ward or aged care facility. Workers are less likely to have a previous relationship with the patient, unlike a family physician or a dialysis nurse. Additionally, patients and associates present to emergency care with already elevated stress levels [11]. In recent years, emergency care usage has increased considerably [12–15]. Patients engage with emergency care more readily for various reasons. The staffing and resourcing of emergency care is not always in line with the increased use of the emergency health services [16, 17].

To design better interventions to prevent and minimize violence, an improved understanding of variations in environments as well as in workers’ approaches to violence is needed.

Previous studies have advocated for the application of a modified social-ecological model to workplace violence prevention efforts. This model presents a series of concentric circles beginning with individual factors in perpetrators or healthcare workers; progressing to factors influencing the relationship between workers and perpetrators; then to factors in the immediate work environment; and finally factors in the wider organization. Identifying risk factors and interventions at each of these levels is important for preventing workplace violence [18–21].

For the prevention of workplace violence, it is equally important to investigate what strategies emergency health care workers use to prevent or minimize workplace violence. There have been numerous studies on emergency health care workers’ experience of workplace violence [1, 4, 22–26], underpinning the frequent occurrence of violence and the severe impact it has on workers. Limited focus has been on strategies used by emergency health care workers to address violence [1, 27] or on the use or impact of these strategies.

In this study the focus is on paramedicine as a distinct work environment. Both paramedics and dispatchers (together referred to as emergency health care workers in this manuscript) from three different jurisdictions in Canada were asked to identify strategies they use in response to violence from patients or bystanders. They were also asked about their experience with these strategies and whether they reduced or minimized workplace violence.

For the purpose of this study, community paramedics were included. Even though they generally do not respond to emergency situations, they do work in a relatively uncontrolled environment, and they usually work alone. These factors introduce a heightened level of vulnerability in violence situations.

The aim of the study was to identify and discuss strategies used by emergency health care workers, in response to workplace violence and aggression. Mapping the strategies used and their perceived usefulness will inform the development of tailored interventions to reduce the risk of serious harm to health care workers.

This study addressed the following research questions:

1. What strategies do prehospital emergency health care workers utilize against workplace violence from patients or bystanders?
2. What is their experience with these strategies?

## Methods

For this study we conducted focus groups with prehospital emergency care workers, using a descriptive qualitative design [28], as the nature of the study was exploratory.

### Study setting

Five focus groups with emergency healthcare workers (paramedics and dispatchers) were held at different locations in Canada: three in Ontario and two in British Columbia. Three groups were in an urban setting and two in a rural setting.

### Study sample and recruiting procedure

The local organisations invited their emergency health care workers, either via email, in meetings, or face to face to participate in the focus groups at a set time, resulting in random sampling. The only inclusion criterium was being an emergency health care worker. The number of focus groups was determined by the number of organisations that agreed to participate. The number of participants was in theory capped at 10–12 to give all participants the opportunity to participate fully. A Participant Information Statement was provided, which explained the purpose of the study, the voluntary nature of participating and the role of the researcher. Potential participants given the opportunity to ask questions and discuss the information with others if they wished.

### Data collection procedure

The focus groups had three to six participants (see Table 1) and lasted a maximum of 90 min. They were audio recorded for transcription and analysis. Written consent was obtained at the start of each focus group that was moderated by ES, a female researcher on the project. No additional persons attended the focus groups. To create an environment that was as optimal as possible for participants to unreservedly articulate their responses and to eliminate any impacting power differentials, all persons with line management responsibility did not attend the focus groups. In addition, there was no relationship between the facilitator/principal investigator and the workers. The facilitator was an Australian researcher, the participants Canadian workers. To improve the dependability of the study, an audit trail was kept by the principal investigator including observation notes and a reflexive journal. The reflexive journal was kept, to assist the confirmability of the study [29].

**Table 1:** Overview of focus group participants

Focus Group	Location	Number of Participants	Female participants	Profession
1	Ontario, Canada	6	0	Paramedics
2	Ontario, Canada	5	0	Paramedics and dispatch
3	Ontario Canada	3	2	Paramedics/ researchers
4	British Columbia, Canada	5	1	Community paramedics
5	British Columbia, Canada	6	3	Paramedics

For the reporting of our results, we used the COREQ standard [30]. Two broad questions were used to explore the research questions for this study: The participants were asked [1] whether they identified different groups of perpetrators of violence and [2] what their approach was based on their assessment. The term perpetrator is used in this study to describe a person who uses violence and is not intended to invoke legal or criminal connotations [31].

### Data analysis

The focus group responses were transcribed verbatim and analysed using a phenomenological approach, as this approach centres around the lived experience of participants [32]. Because of the exploratory nature of the study, inductive thematic analysis [33]; was used as it allowed themes to emerge from the data without the analyst searching for specific answers, which would have been more in line with deductive analysis [34]. The data were coded by ES and JV.

Ethical approval.

Ethical approval was granted by La Trobe University Ethics Committee under number HEC19009 and by the Hamilton Integrated Research Ethics Board, project 7031.

This ethical approval process was approved and supported by the REBs of the paramedic services involved.

## Results

### Participants

The five focus groups comprised of 25 participants in total (Table 1). They had been working in the field for an average of 13 years (range 5 – 38 years). There was a gender imbalance in the participants sample, with only six female participants, which is reflective of the workforce in this setting [9, 35, 36]. No participants dropped out of the focus groups. Participants had differing opinions across and within focus groups, resulting in lively and unreserved discussions in which everyone expressed their opinions and experiences.

## Strategies and experiences

With thematic analysis, six major themes on strategies to prevent and deal with violent behavior were identified: training and other tools, support for refusal of care/staging, prevention strategies, communication between organizations, flagging, and dispatch. One additional minor theme was identified that did not fit within the six major themes: the uniform. The themes are presented and discussed below and supported with quotes in the text and in Table 2.

**Table 2:** Overview of themes supported with quotes

Theme 1: Training/Tools
•But I would also like to see some policy – some training, because we have very minimal training in diffusion. I’m not looking for self-defense stuff, because we shouldn’t be fighting. That’s not my issue.” Participant Y •I would go back to the idea of having training on how to defend ourselves. How to disarm a patient or situation. Actual hands-on self-defense training. It shocks me that we don’t have any at all, really. Participant C •There have been some requests for different self-defense training and things like that, that were pushed back in favor of ‘No, we’re just going to focus on de-escalation and reporting.’” Participant P •You still don’t have to attack a paramedic. But the intervention is focused on it’s the paramedics’ fault that they are assaulted. Participant B •And I guess just after a couple of years on the job I realized that if the 911 call doesn’t involve them, I can ask them to leave. I can’t necessarily ask the patient to go away because they called 911. They’re in medical distress. But anybody who doesn’t need to be involved, I can ask them to leave. And ask the police to say if they won’t leave, you need to arrest them and get them out of here. Participant U •De-escalation, all that kind of stuff. I find that we don’t have a lot of that. And we deal with a lot, like a lot of violence. Participant Y •We also have checks in place whereby we contact the family before each visit, a phone call. And we have a checklist that we go through. And that’s done each time before we attend that resident. Participant R •I think for us as community paramedics, everybody is – we have a big responsibility for our own safety. We’ve been given the tools. We just have to use them. […] I mean number 1 is remove yourself from the situation. Participant R •Most of CPs now have public event kind of thing, like a wellness clinic. And that’s where we invite people to come see us if they want to see us if we have any concerns at all. And that way we’re never in a situation where we might be caught alone with them or where there is any threat to us. Participant Q Sedation / restraints / spit hoods •There is a big push for that, and everyone is very proud of the fact that they don’t restrain patients chemically or physically. Participant X •I had a call recently, and the guy was being aggressive. The police didn’t want to do much, but my partner got an order to sedate the patient, and that made everything go smooth on the call. No danger. Participant Y •And then, in terms of what people want in the future, there is a lot of equipment, like spit-hoods, and soft restrains that are available to us, but we’re not using them here. Participant S
Theme 2: Support for refusal of care / staging
•I also don’t think we’re terribly supported by the act that governs us. So, the ambulance act is very, very limited capability for paramedics to refuse going in to help somebody. Participant K •And I guess the problem is, what if that patient has had an opioid overdose, and they’re slowly decreasing respiratory. And that’s the balance on the other side, there is a potential hazard for that patient. That’s the balance. Participant X •I mean number 1 is remove yourself from the situation. And that’s been spoken to very well by everybody. That’s the ultimate solution for us. Participant R •So, you’re constantly saying is my job worth potentially staging, is my safety worth potentially saving, and it’s that argument and that call. Participant V •If I walk in and someone says, ‘Fuck off’, I want the ability to just say ‘Okay, see you later. I’m not helping you.’ It’s never going to happen. And that’s not what we signed up for. But if they’re going to treat us badly, then I want the ability to just walk away and say an ambulance is not going to be helping you. Participant U •Because we’ve had calls, there was a call in [city], where the paramedics didn’t feel safe. Didn’t enter the home. And the patient died as a result. And those paramedics ended up being punished pretty severely for that. So, the whole idea, it’s our discretion on scene, is sort of a grey area. Participant R •Here actually, if a paramedic calls dispatch and says ‘Hey, I’m just not going. I’m going to stage.’, they’re actually really supportive. Participant K •We’re required to enter a potentially unsafe scene and only leave if there is explicit evidence of danger. Participant T
Theme 3: Prevention strategies
•So, whether it’s us being a little – meeting with hospitals and actually developing a transportation policy for these patients. You will always send an escort. They will always have medication available to sedate the patient, etc. That sort of thing. I think those are the sort of things that we can be proactive on. Participant X •I think more public education could really help too. I’ve seen the videos that they’ve put out in Australia. Participant I •If you get on the bus in [city], there is a little sign that says, ‘Assaults on the bus driver, whoever, blah blah blah, will not be tolerated.’ I’m never seen such a sign in an ambulance. Participant C •I think advertising is certainly a part. We want to shape the public conversation around this and want to let people know that the behavior is not acceptable. Participant T •There is a discussion around putting those stop signs in the back of ambulances. And I’m actually against it, personally. Because you see you got them in Australia, and it hasn’t done anything. […] Threats have to be immediate, realistic and enforceable. If we say, violence threats will not be tolerated, you will be kicked out the ambulance. Somebody reads that and goes ‘Okay, make me.’ Participant P
Theme 4: Communication / Information sharing
•I look at it in a different way to, that unfortunately we get too much information before we go stage. They should just tell us it’s a violent incident. Shouldn’t tell us anything about – shouldn’t tell us where it is. They should give us a general area to stay in, take the human factor out. Participant Y •All I know it’s a horrible system where the police have so much information than us on every address. Participant Y •One of the biggest places there where we see a gap is in information sharing. […] I think in lot of cases there is not sufficient information transferred from a sending facility in regard to the patient. Participant R •But there has been really inconsistent action taken by dispatch. And dispatch is not employed by us. They’re a different entity. So you can’t really control what they actually do. Participant S •They’re our first line of defense. Dispatchers. Participant S •Generally speaking, when we go to a place, like a known drug dealers house or something that police are aware of, they’ll let us know. And usually they’ll say don’t enter the building until police have arrived. Participant V •I think we have the same issues that everyone in the world does. We feel the information is often incomplete. And that doesn’t allow us to complete an accurate risk assessment of the scene. And that is not in any way a dispatcher’s issue. Participant N •Here actually, if a paramedic calls dispatch and says ‘Hey, I’m just not going. I’m going to stage.’, they’re actually really supportive. Participant N •Yeah, police called us for this. And then they’re not on the road. Why are we going? Particpant U •We’ll show up for domestic disputes before police. Participant I •Same thing, we used to always be – when I worked in [city], we were the first call for all dropped 911 calls. And I’m like ‘Why are we doing dropped 911 calls?’ Participant Y
Theme 5: Flagging
•They can flag, but the process to flag is not very… […] it expires in a year. Participant Y •It’s usually a repetitive thing. Like 3 or 4 times we’ve been to this address and every time we’re dealing with it. It will eventually pop up. And sometimes police will have flags, that we don’t know about. So they contact [dispatch]. Participant V •So, one of the things that we historically had trouble with is reluctance from some parts of the organization to what we call as flagging an address. Participant P •It’s not common practice. It’s difficult – it’s not super difficult to do. But there are institutional or bureaucratic processes to try and discourage it, I would say. Because nobody wants to take the responsibility if someone moves and then we don’t go in and grandma dies. Participant P
Theme 6: Dispatch
•Because they’ve had a conversation with the people on scene already. And nobody else has at that point, right? They’re the only ones that have that information. So there is a trust factor that has to exist between our dispatch and us. Participant V •So for us – we’re not dealing with face-to-face violence, but you’re observing that. And we get attacked verbally. Participant M •There is no riding third man in Dispatch. There is no option to know this kind of call is going to trigger me, so I’m not going to take any of those kind of calls. You don’t know. Before you answer, you have no idea what you’re getting into. Participant M
Uniform
•We dress very similar to the police. […] A lot of people misunderstand that we’re there to help medically. We’re not there to charge and do that. Participant V •I find also, us looking some much like police officers has given me issues in my career. I’ve been attacked because we look so much like cops. Participant Y •Yeah, I think generally on the downtown east side paramedics are viewed favorably. […] I do feel like I’m in a bit in a suit of armor when I’m in my uniform down there. Participant C

### Training and other tools

Participants had mixed views on the use and usefulness of training. Some participants would like to get more self-defense or de-escalation training, as this appeared to support them in dealing with violent incidents and made them feel more in control. Others did not think more training would be helpful and even felt this should not be part of their job, as their role was delivering health care, not engaging in the resolution of violent situations. The training could put too much responsibility on the health care worker. There is a risk of making violence incidents their responsibility and even their fault if the violence got out of hand and it was believed they could have done more to resolve it. At the same time, the workers felt they were the victim of the violence. They felt that the organization could take more responsibility, for example there was no clear policy in place on how to deal with violent perpetrators. Irrespective of the training they might have had, most participants mentioned having their own strategies when dealing with violent behavior. They acknowledged that their approach varied, depending on their assessment of the type of perpetrator.

Restraints and sedation were also mentioned as tools to deal with violence. Paramedics saw (soft) restrains as useful but were skeptical that management would allow them.

*“But I would also like to see some policy – some training, because we have very minimal training in diffusion. I’m not looking for self-defense stuff, because we shouldn’t be fighting. That’s not my issue.”- Participant Y *

“And I'm not even sure of the actual reporting process for violence in the workplace. To be completely honest with you" - Participant H

### Support for refusal of care /staging

Support for refusing the patient health care was a second theme. Most focus groups participants mentioned that they would like to be allowed to refuse the patient care and avoid entering a violent situation. Being allowed to refuse care was seen as clear organizational support for the worker and would contribute to a safe workplace, however participants had doubts this was possible. Regardless of this, some participants did mention they had walked away from violent situations to protect themselves.

*“And to have an institutional sort of, even if it is purely theoretical, back-up to my position of saying ‘Sorry, but you’re going to have to leave my ambulance. I’m not going to take you to the hospital today.’ That would go a long way I think. To be supported in that decision.” – Participant C*

For paramedics, ‘staging’ outside a patient’s home until police arrived was another clear way to ensure safety. The principle of staging is to keep paramedics safe until police secure a (potentially) violent scene. The paramedics park out of view, leave themselves a buffer (stay out of range) and only respond when the police have reported the scene is secure, making this different to a refusal of care. When a scene suddenly becomes dangerous and paramedics need to retreat, the same principles should be applied. Participants to the focus groups experienced a range of different levels of support from their organization and from dispatch to do this. Dispatch refers to the central staff who receive emergency calls, provide essential pre-arrival advice, and coordinate and dispatch resources and patient transport movements.

Focus groups participants felt supported by dispatch in their decision to stage.

Staging appeared to be an acceptable practice and paramedics agreed that this made them feel safer. However, looking at the bigger picture, participants mentioned that a grey area exists between liability and safety, with the liability potentially falling on the worker in the event that something goes wrong. In common with remarks in relation to training, it appears that workers feel vulnerable on a number of levels: they feel liable, feel they are made responsible, and do not feel sufficiently supported by their organization or by clear policies.

Participants felt a strong duty of care. Besides their own personal safety, participants felt a conflict between staging and the patient’s safety and their duty of care. They mentioned having to find a balance between the two. For workers, this can be described as moral distress.

“So, you’re constantly saying is my job worth potentially staging, is my safety worth potentially saving, and it’s that argument and that call.” – Participant V

“Here actually, if a paramedic calls dispatch and says ‘Hey, I’m just not going. I’m going to stage.’, they’re actually really supportive.” – Participant N

*“If there is an immediate and obvious danger – we all just hold off. Sometimes – it’s the balance that you’re talking about. If there is someone going downhill on the other side of that door, and you know it – what are the risks versus you know –” –  Participant W*

### Prevention Strategies

The focus groups were generally not convinced that advertising or public campaigns to prevent violent incidents were useful. Some participants saw it as a way to shape the public conversation and to educate the general public, noting that the problem extends beyond their workplace. Others did not see it as the solution, they did not believe it was enough to deter people, they did hope that it could increase awareness around the issue. Finally, participants felt it would not change the risk and might even induce patients to become more violent. They felt that saying violence threats will not be tolerated, as some campaigns do, without actually following up on this zero-tolerance strategy, would encourage people to push the boundaries. This lack of action made them feel more vulnerable.

*“We prepared a public awareness campaign. […] I mean I don’t think that public advertising is going to prevent violence by any means. But at least they’re aware and it might target people who are just in a stressful situation and are more verbally abusive or harassing or threatening to paramedics.” –   Participant S*

*“There is a discussion around putting those stop signs in the back of ambulances. And I’m actually against it, personally. Because you see you got them in Australia, and it hasn’t done anything. […] Threats have to be immediate, realistic and enforceable. If we say, violence threats will not be tolerated, you will be kicked out the ambulance. Somebody reads that and goes ‘Okay, make me.’” – Participant P*

### Communication between organizations

Communication between organizations was a fourth identified theme. Participants commented mostly on a lack of information provided to them which could create unnecessary risks. For example, between hospitals, flagging, discussed in more detail below, was not communicated.

Participants felt that more information from dispatch or the police could help with decisions around staging and an accurate risk assessment of the scene. Others preferred to know less when staging, as it reduced the risk of moral distress. Again, this theme was related to the responsibilities of a paramedic. Some participants felt that they were called to emergency where the police should have been first on the scene.

Participants mentioned very good collaboration with their dispatchers, who they saw as very positively contributing toward their safety. Dispatch was seen as diligent and proactive to ensure that the paramedics were safe and were provided with complete information.

A final issue around communication came from the community paramedics, who mentioned that there is often insufficient information in relation to a transport patient; information can help to identify preventable risk.

*“And it’s a difference if we have more information from dispatchers. Did she decide to pick up the phone and call for help? That’s a different mentality, because now they are asking for help versus somebody interrupted their plan. Now they’re angry. So, we don’t have access to that.” – Participant Y*

“All I know it’s a horrible system where the police have so much [more] information than us on every address.” – Participant Y

“Generally speaking, when we go to a place, like a known drug dealers house or something that police are aware of, they’ll let us know. And usually they’ll say don’t enter the building until police have arrived.” – Participant V

“The call takers are really diligent at trying to work out if there is any risk for us. Which I appreciated.” – Participant B

“One of the biggest places there where we see a gap is in information sharing. […] I think in lot of cases there is not sufficient information transferred from a sending facility in regard to the patient.” – Participant R

### Flagging

Flagging was identified as a distinct organizational theme which is why we identified it as a separate theme. It is a strategy to signal repeat offenders. Flagging means that if a specific patient calls for paramedic help or presents to a hospital, it should alert the emergency health care worker, if they have been known to have been previously violent. One service, which had the dispatch as part of the service, felt that this was useful, as it allowed the dispatchers to inform paramedics of known or documented dangers associated with a given address. But other participants mentioned that it was a difficult process to get in place, often relating this to reluctance from the organization to allow flagging as a strategy.

“What we do tend to do with our dispatch is that if we’ve gone to a location where we had a violent encounter, we’ll flag that with our dispatchers so future calls they can let them know that this patient has a history of violence against emergency personnel.” – Participant V

“So, one of the things that we historically had trouble with is reluctance from some parts of the organization to what we call as flagging an address.” –   Participant P

### Dispatch

Two elements within the theme dispatch were identified. The central role of dispatch emerged as important with the paramedic participants. When communication and collaboration with dispatch were good, dispatch was seen as ‘the first line of defense’. They were seen as being able to provide crucial information, because they had a conversation with the people on the scene. As a second element, in our study, it also became apparent that dispatchers deal with a lot of verbal violence. It was mentioned that since dispatchers do not get physical injuries, their exposure to violence may not be fully acknowledged and nothing is necessarily going to keep them from coming back, even though they might have been affected mentally. Integrating back into dispatch was considered to be challenging, as dispatchers cannot assess beforehand, when they accept a call, what is on the other end of the line.

“Because they’ve had a conversation with the people on scene already. And nobody else has at that point, right? They’re the only ones that have that information. So there is a trust factor that has to exist between our dispatch and us.” – Participant V

“So for us – we’re not dealing with face-to-face violence, but you’re observing that. And we get attacked verbally.” – Participant M

“There is no riding third man in Dispatch. There is no option to know this kind of call is going to trigger me, so I’m not going to take any of those kind of calls. You don’t know. Before you answer, you have no idea what you’re getting into.” – Participant M

### The uniform

The paramedics uniform was seen as an issue by some of the participants as in one province they look very similar to police. This could sometimes cause potential danger and inhibit the paramedic’s capacity to provide care. Others mentioned that their uniform gave them a, what they saw as, false sense of security as paramedics were highly respected in the area.

## Discussion

### Main findings

This study focused on the use of strategies to deal with violence and the experience with these strategies. Participants identified several commonly used strategies to prevent and minimize violence from patients and bystanders, that are utilized by emergency health care workers. While all participants identified similar strategies, there were opposing views in relation to all strategies, often relating to their experienced usefulness, appropriateness, and legitimacy.

The results show that emergency or first responder work environment is distinctly different to the more controlled environment of a hospital ward when it comes to both exposure to violence, and the capacity to reduce and minimize violence. While the study samples were small, they were diverse and included both urban and rural settings. This paper reiterated that violence has a serious impact on emergency health care workers, to the point where some workers would like to have the option to refuse care. This is a very strong indication of how serious this problem is, as it has already been identified that the duty of care weighs very heavily on all health care workers [9].

### Interpretation of the findings

The results of this study highlighted several issues around violence at work that are discussed more in-depth below.

### The social- ecological model of workplace violence

The social-ecological model of workplace violence [21] was a fitting framework for our results. The model aligned well with the themes identified through our analysis. The clearest indication appeared to be that, when applying the results to the model of workplace violence, most interventions fit within the work environment level and participants felt the responsibility sits at the individual level. At the same time the participants were seeking support and clarity around interventions at the organizational level. A suggestion for consideration is to include an overarching ‘societal level’ to the model, as this type of workplace violence has a strong societal component [37–39]. Examples of interventions at this level were the public campaigns. And although these campaigns were not considered to be very effective, participants did agree on the importance of raising workplace violence as an issue with the general public.

### Strategies and experiences

The focus group participants identified a variety of different strategies based on what they feel is available or has evolved over time as seemingly useful; often these options are more readily based on anecdote than evidence. The suggested strategies appeared to be specific for an emergency health care setting: e.g., staging, flagging and collaboration with the police. This differs from the standard strategies used in other settings, for example psychiatric nurses, would focus on risk assessments and de-escalation techniques [40]. Many participants discussed the value of education and training, however they stressed that this should simply be one component that complements a system of violence prevention. As mentioned in the introduction, training is often seen by organizations as a one size fits all tool to prevent violence. In this study, participants saw training as a tool for the worker to keep themselves safe and to prevent violence. Although participants had mixed views about extending the training to self-defense training. While participants discussed the value of training, a second recent Cochrane review found that, while training and education may result in a possible increase of personal knowledge and positive attitudes, it did not have an effect on workplace aggression directed towards health care workers [41].

### System-based approach to violence

The study results indicate that, when looking at the different levels of violence, dealing with violence must be integrated into the organization, rather than being made the individual responsibility of the worker, who is in essence the victim in this situation. Most of strategies identified, such as flagging, staging and collaboration, reflect a use of the system, as opposed to making the individual worker (the victim) responsible for an adequate approach through training. This system-based approach must be addressed at the organizational level, providing clarity on policies and procedures. In addition, effective collaboration outside of the health system is reliant on clear roles and responsibilities. For example, the participants had differing views on their collaboration with the police. Some stated that paramedics are sometimes called to a situation that the police should visit first, given their broader authority to act. This is interesting as the trend for mental health calls is to reduce reliance on police. In many parts of the world paramedics are teaming up with social workers or mental health nurses to respond to mental health calls [42, 43]. Attention to the types of perpetrators may support the organization of these responsibilities.

### Importance of dispatch

Dispatchers play a pivotal role in the system of violence prevention for paramedics. Dispatch could provide relevant information and was a point of contact to discuss and even legitimize an approach to a situation. The results highlighted that dispatchers themselves are subject to (verbal) violence with little recourse. Return to work is challenging as they do not know what situation they might land themselves in before they pick up the phone, there is no buffer. In addition, their exposure rate is significantly higher than paramedics. Whereas a paramedic may do 6 to 8 calls in a standard 8-h shift, a dispatcher may take that many in an hour, often in rapid succession. In addition, research has shown that verbal assault is the most common form of violence [1]. Call-taking and dispatching has been described as invisible work; the stressful nature of their work needs to be recognized with adequate support [44–46].

### Establishing systems of support

The emphasis on systems, organizations, and collaboration was present in many responses, including accepting the usefulness of public campaigns, if these are followed up with a consistent and consequent system response. Any response to violence needs to be part of a support system at the organizational level and needs to be ratified and backed by the organizations involved, not only by a hospital or health care organization but also by the professional and industrial bodies of emergency health care workers. Commitment and endorsement of management is as important to the effectiveness of workplace violence prevention as the prevention strategies themselves [47, 48]. A lack thereof can seriously impact the health and well-being of the worker and act as a barrier to workplace violence prevention [49]. 

### Strengths and limitations

While the study samples were small, they were diverse and included both urban and rural settings. The study is not supported by quantitative data, but the qualitative approach provides deeper insight into the experiences of pre-hospital workers with violence. In addition, the results aligned well with the social-ecological model of workplace violence. The prehospital perspective compliments similar research conducted in emergency departments.

## Conclusions

This study demonstrates that emergency health care workers use a variety of strategies when dealing with violent patients or bystanders. Most strategies, other than generic de-escalation techniques, reflect a reliance on the systems the workers work with and work in. This supports the move away from focusing on the individual worker, who is the victim, to a systems-based approach at the organizational level to help reduce and minimize violence against health care workers. For this to be effective, system-based strategies need to be implemented and supported in health care organizations and legitimized through professional bodies, unions, public policies, and regulations.

## Data Availability

The datasets generated and/or analysed during the current study are not publicly available due to privacy restrictions but are available from the corresponding author on reasonable request.
